# Activating transcription factor (ATF) 6 upregulates cystathionine β synthetase (CBS) expression and hydrogen sulfide (H_2_S) synthesis to ameliorate liver metabolic damage

**DOI:** 10.1186/s40001-023-01520-w

**Published:** 2023-11-25

**Authors:** Bingzi Dong, Ying Sun, Bingfei Cheng, Yu Xue, Wei Li, Xiaofang Sun

**Affiliations:** 1 Department of Endocrinology and Metabolic Diseases, The Affiliated Hospital of Qingdao University, Qingdao, 266003 China; 2 Health Management Center, The Affiliated Hospital of Qingdao University, Qingdao, 266003 China; 3 Interventional Medical Center, The Affiliated Hospital of Qingdao University, Qingdao, 266003 China

**Keywords:** Activating transcription factor 6 (ATF6), Cystathionine β synthetase (CBS), Sulfhydration, Non-alcoholic fatty liver disease (NAFLD), Hydrogen sulfide (H_2_S), Inflammation

## Abstract

Activating transcription factor 6 (ATF6) is an endoplasmic reticulum stress responsive gene. We previously reported that conditional knockout of hepatic ATF6 exacerbated liver metabolic damage by repressing autophagy through mTOR pathway. However, the mechanism by which ATF6 influence liver metabolism has not been well established. Hydrogen sulfide (H_2_S) is a gaseous signaling molecule that plays an important role in regulating inflammation, and suppress nonalcoholic fatty liver in mice. Based on the previous study, we assumed that ATF6 may regulate H_2_S production to participate in liver metabolism. In order to clarify the mechanism by which ATF6 regulates H_2_S synthesis to ameliorate liver steatosis and inflammatory environment, we conducted the present study. We used the liver specific ATF6 knockout mice and fed on high-fat-diet, and found that H_2_S level was significantly downregulated in hepatic ATF6 knockout mice. Restoring H_2_S by the administration of slow H_2_S releasing agent GYY4137 ameliorated the hepatic steatosis and glucose tolerance. ATF6 directly binds to the promoter of cystathionine β synthetase (CBS), an important enzyme in H_2_S synthesis. Thus, ATF6 could upregulate H_2_S production through CBS. Sulfhydrated Sirtuin-1 (SIRT1) was downregulated in ATF6 knockout mice. The expression of pro-inflammatory factor IL-17A was upregulated and anti-inflammatory factor IL-10 was downregulated in ATF6 knockout mice. Our results suggest that ATF6 can transcriptionally enhance CBS expression as well as H_2_S synthesis. ATF6 increases SIRT1 sulfhydration and ameliorates lipogenesis and inflammation in the fatty liver. Therefore, ATF6 could be a novel therapeutic strategy for high-fat diet induced fatty liver metabolic abnormalities.

## Introduction

Endoplasmic reticulum (ER), as a vital membranous organelle, is closely involved in protein synthesis, folding and processing. However, research has verified that numerous metabolic stimuli and nutritional changes can contribute to the accumulation of unfolded and misfolded proteins, and finally result in ER stress by activating the cellular unfolded protein response (UPR) [1]. The liver, a predominantly metabolic organ, possesses abundant ER. Obviously, activation of the UPR in the liver plays a vital role in the regulation of glucose and lipid metabolism [2, 3].

Activating transcription factor 6 (ATF6), a transduction molecule in the downstream signal pathway of UPR, combines to the ER stress response element (ERSE) to regulate the homeostasis of ER and maintain cell functions. Previous studies showed that ATF6 not only inhibited gluconeogenesis, but also attenuated hepatic steatosis through interacting downstream regulators [4–6]. Our previous studies have reported that conditional knockout of hepatic ATF6 exacerbated liver metabolic damage mainly through mTOR pathway, which suggested that ATF6 may be a potential target for the remission of liver metabolic impairment [7]. However, the exact mechanism by which ATF6 affects the high-fat diet induced fatty liver metabolism is not well established.

Hydrogen sulfide (H_2_S), as an important gasotransmitter, is involved in various physiological and/or pathological processes including glucose metabolism and lipid synthesis ascribing to the formation of protein persulfides or protein S-sulfhydration [8–10]. The endogenous H_2_S is mainly generated by cystathionine β-synthase (CBS), cystathionine γ-lyase (CTH) and 3-mercaptopyruvate sulfurtransferase (MST), all of which are present in the liver [12]. Hepatic H_2_S metabolism dysfunction may disturb the liver metabolism and cause liver diseases [10, 11]. In the liver, a rich source of CBS involved in the H_2_S homeostasis [12]. It is reported that CBS is crucial to maintain the normal liver function [13]. CBS deficiency results in liver disorders by affecting the H_2_S function in hepatocytes under stress conditions [14]. Besides CBS, CTH together with H_2_S have been reported to be associated with hepatic lipid metabolism [15]. SREBPs (sterol regulatory element binding transcription factors) are master transcriptional proteins that regulate the expression of genes associated with lipid homeostasis. SREBP1 directly suppresses the expression of CTH, one of the main metabolic enzymes responsible for synthesis of H_2_S in the liver, and downregulates the ULK1 sulfhydration, blocks the autophagosomes with lysosomes and promotes hepatic steatosis [16]. SIRT1 is involved in the regulation of lipid and glucose metabolism in the liver, and associated with the development of NAFLD [17]. Zhou et al. reported that Resveratrol stimulated SIRT1 expression significantly, and increased deacetylation and inactivation of ATF6, and ameliorates lipid droplet accumulation in liver through SIRT1/ATF-dependent way [17]. However, the mechanism how H_2_S regulates liver lipid metabolism remains unclear.

Based on the previous study, we assumed that ATF6 may regulate H_2_S production to influence the liver metabolism, and conducted further investigation. In addition, the mechanism by which ATF6 regulates H_2_S synthesis to ameliorate liver steatosis, and the role of CBS and inflammatory factors in liver metabolism remains uncertain. In the present study, we demonstrated that H_2_S level was significantly downregulated in hepatic-specific ATF6 knockout mice, leading to hepatic steatosis and glucose tolerance. ATF6 directly binds to the promoter of CBS, and transcriptionally enhance CBS expression as well as H_2_S synthesis. Thus, we illustrated that ATF6 increases SIRT1 sulfhydration by H_2_S production, and ameliorates steatosis and inflammation in the fatty liver. Therefore, ATF6 could be a novel therapeutic strategy for high-fat diet induced fatty liver metabolic abnormalities.

## Materials and methods

### Animals, cell lines and plasmids

#### Animals

The C57BL/6J mice, B6(Cg)-Atf6tm1Hota/J mice and B6/JNjuAlbem1Cin(icre)/Nju mice were generated. The liver specific ATF6 knockout mice (hereinafter referred to as ATF6^−/−^ mice) were generated by crossing B6(Cg) Atf6tm1Hota/J mice and B6/JNjuAlbem1Cin(icre)/Nju mice as previous reported [7]. All animals were housed and maintained on a 12 h light–dark cycle and on a regular unrestricted diet and free access to water. The male wildtype (WT) and ATF6^−/−^ male mice were littermates and randomly selected. Since 8-week-old, the male mice were fed with high fat diet (HFD, 45% fat) for 8 weeks. At 8-week-old, there was no difference of the body weight among groups. At the beginning, the body weight in different groups were as follow, WT + HFD 25.86 ± 0.55 g, ATF6^−/−^ + HFD 26.32 ± 0.65 g, and ATF6^−/−^ + HFD + GYY 26.09 ± 0.84 g, (*p* > 0.05). The mice were administered with GYY4137 at 50 mg/kg/day by intraperitoneal injection for 4 weeks. Mice were fasted for 16 h, then scarified for serum and liver samples collection. All animal experiments were conducted under protocols approved by the Animal Research Committee of the Affiliated Hospital of Qingdao University. The agent of GYY4137 (CAS 106740-09-4) was commercial available and purchased from Santa Cruz Biotechnology (Dallas, Texas, US).

### Analytical procedures

We used a glucometer (One Touch Ultra; LifeScan, Milpitas, CA, USA) to detect the blood glucose using capillary blood samples from mice tails. Serum and liver concentrations of triglyceride and cholesterol were collected after 16 h fasting, and detected by an automated Monarch device (The Affiliated Hospital of Qingdao University, Shandong, China).

### Glucose and insulin tolerance test

Intraperitoneal Glucose tolerance test (ipGTT) was carried out after 16 h fasting. The mice were given glucose (1 g/kg) by intraperitoneal injection. The blood glucose was detected using glucometer at 0 min, 15 min, 30 min, 60 min, 90 min and 120 min. Insulin tolerance test (ITT) was conducted after 6 h fasting. The mice were given insulin (0.75 IU/kg) by intraperitoneal injection. Blood samples were collected and glucose levels were measured at 0 min, 15 min, 30 min, 60 min, 90 min and 120 min.

### Histology

Liver tissue was extracted and fixed in 4% paraformaldehyde, then processed it for staining of paraffin-embedded sections. For histological evaluation, Hematoxylin–eosin (HE) staining was performed at the Central Research Laboratory, The Affiliated Hospital of Qingdao University following standard procedures.

### Primary cell culture

Primary mouse hepatocytes were isolated from livers of male WT and ATF6^−/−^ mice after 8 weeks HFD-feeding following the protocol. Briefly, mice were anesthetized, and livers were perfused with 0.5 mg/mL type II collagenase (Sigma–Aldrich) via the inferior vena cava to isolate hepatocytes. Primary hepatocytes were cultured in RPMI-1640 with 100 units/mL penicillin, and 0.1 mg/mL streptomycin, with serum free medium for the following investigation.

### Cell lines

HepG2 cells were kind gifts from Professor Xiaopan Wu (State Key Laboratory of Medical Molecular Biology, Institute of Basic Medical Sciences, Chinese Academy of Medical Sciences, School of Basic Medicine, Peking Union Medical College, Beijing, China). HepG2 cells were incubated in Dulbecco’s modified Eagle’s medium (DMEM) with 10% fetal bovine serum (FBS), and were maintained in 5% CO_2_ at 37 °C.

### Plasmids and transfections

ATF6 overexpression plasmids and CBS promoter PGL3 plasmids were kind gifts from Professor Xiaopan Wu (State Key Laboratory of Medical Molecular Biology, Institute of Basic Medical Sciences, Chinese Academy of Medical Sciences, School of Basic Medicine, Peking Union Medical College, Beijing, China). ATF6 overexpression plasmid was described as previously reported [18]. CBS promoter plasmid was constructed by Shanghai Generay Biotech Co., Ltd (Shanghai, China). The sequence of binding site of upstream promoter was predicted using Alibaba 2.1 (http://gene-regulation.com/pub/programs/alibaba2/index.html). Transfection experiments were performed using Lipofectamine 3000 (Thermo Fisher) according to the manufacturer’s instructions.

### Quantitative PCR

According to the manufacturer’s instruction, total RNA was extracted from the liver tissues and HepG2 cells by TRIzol reagent (Invitrogen, USA), and it was reversed transcribed into the cDNA in a 10 μL reaction volume by ReverTra Ace qPCR RT Master Mix (TOYOBO, Japan). The primers were synthesized as the report [19, 20], and the sequences of primers were listed as bellow. Mouse GAPDH-F, CAGCAACTCCCACTCTTCCA, GAPDH-R, CATGAGGTCCACCACCCTGT. Mouse ATF-6 F, GCCAGACTGTTTTGCTCTCT, ATF-6 R, CTGTCTTTCTGGTTGTCACC. CTH-F, TTGGATCGAAACACCCACAAA, CTH-R, AGCCGACTATTGAGGTCATCA. CBS-F, GCCATCAGACGAAGTCTGCAA, CBS-R, TGGTCCATCTCCAGGATGTGA. SREBP1c-F, GCGCAGATCGCGGAGCCAT, SREBP1c-R, CCCTGCCCCACTCCCAGCAT. The relative expression of targeted genes was normalized to internal control GAPDH and analyzed by the comparative Ct method.

### Protein sulfhydration

The protein extracts were homogenized into RIPA lysis buffer, and 4 mL of blocking buffer was added (containing 225 mM HEPES–NaOH, pH 7.7; 0.9 mM EDTA; 0.09 mM Neocuproine; 2.5% SDS; 20 mM MMTS). Samples were kept at 50 °C for 20 min followed. Then the proteins precipitated out in 20 mL pre-ice acetone and were centrifuged for 5 min at 3100 rpm. After that, the pellets were washed with 70% acetone 2 times at room temperature, and resuspend them into HENS buffer (H250 mM EPES–NaOH, pH 7.7; 1 mM EDTA; 0.1 mM Neocuproine; 1%SDS) and 0.8 mM biotin-HPDP. The suspension liquid was rotated for 90 min at room temperature in dark, then the streptavidin resin (BBI, C006390-0005) was added into it and rotated overnight at 4 °C. The streptavidin resin was washed with HENS buffer and then centrifuged for 4 times at 4 °C for 5 min at 2500 rpm. The levels of protein in the samples were detected by western blotting.

### Western blotting

The extracted proteins were separated by SDS-PAGE (1%) and transferred to a polyvinylidene fluoride membrane (Millipore, USA). After blocking with 5% non-fat milk in TBST at room temperature, the PVDF membranes were incubated with primary antibodies, anti-GAPDH (CWBIO, China), anti-ATF6 (Affinity, UK), anti-CBS (Santa Cruz, CA), anti-SIRT1 (Cusabio, China) overnight at 4 °C, followed by the incubation with the IgG (CWBIO, China) at room temperature for another 1 h. Micrographs were taken by the Tanon 5200 Multi (Shanghai, China).

### Dual luciferase assay

HepG2 cells were seeded in 24-well plates and cultured for 24 h. The PGL3 plasmids without CBS promoter were used as the negative controls. Cells were co-transfected ATF6 expression vectors/empty vectors and CBS promoter PGL3 plasmids, and then harvested after 48 h. The luciferase signals were detected using Dual-Luciferase Reporter Assay System (Promega, USA) according to the manufacture’s protocol.

### Electrophoretic mobility shift assay (EMSA)

According to the manufacturer’s instruction, EMSA was carried out by the Chemiluminescent EMSA Kit (Beyotime, GS009). The *CBS* promoter probe sequence was as below: agccttcatgatgtaactccatcc.

### Chromatin immunoprecipitation (ChIP) assay

ChIP was carried out by EZ ChIPTM Chromatin Immunoprecipitation Kit (Merck, 92590) according to the manufacturer’s instruction. The cells were cross liked with formaldehyde. Subsequently the samples were subjected to sonication, and then incubated with anti-ATF6 antibody. The primers used for q-ChIP assay were as follow: sense, 5′-GACAGTCTCGCTCAGTCGCA-3′ and antisense, 5′-GAGGGGACAGGGATGGAGTT-3′.

### H_2_S production measurement

#### H_2_S production measurement in cells

The H_2_S production was measured as previous reported [21]. We placed the microporous filter membrane with 1% zinc acetate on the inner side of the 12-well plates, and then washed the cells 3 times with PBS and cultured them in serum-free medium, in addition with L-cysteine (Santa Cruz Biotechnology, 168149) to normalize the concentration as 2 mM and 5-pyridoxal phosphate (BBI, PD0455) as 0.5 mM. The cells were cultured for 24 h at 37 °C with 5% CO_2_, followed by putting the filter in a test tube with addition of 3 mL ddH2O, 0.5 mL 0.2% *N*,*N*-dimethyl-pphenylenediamine-dihydrochloride solution (2.5 mM) and FeCl_3_ (3.3 mM) (Santa Cruz Biotechnology, F3629). Then the absorbance was detected at 670 nm by the microplate reader (Synergy H1; BioTek; Winooski, VT, USA). H_2_S measurement was calculated according to the standard curve, and presented as nM/(min 10^6^ cells).

#### H_2_S production measurement in the liver of mice

The liver tissues (200 mg) were homogenized in the ice-cold passive lysis buffer (500 µL), and 100 µL homogenates were add into the 12-wells plates, together with 900 μL pre-cold PBS containing 5 mM L-cysteine and 2 mM pyridoxial 5-phosphate into it. The filter membrane soaked in 1% (v/v) zinc acetate solution was affixed to the inner side of the cover, and kept incubating for 24 h at 37 °C in a 5% CO_2_ atmosphere. The filter paper was put into centrifuge tube, and 3 mL ddH_2_O, 0.5 mL 0.2% *N*,*N*-dimethyl *p*-phenylenediamine solution, 50 µL 10% ammonium ferric sulfate were added and mixed. OD values were detected at 670 nm, and H_2_S measurement was calculated according to the standard curve, and presented as nM/(min 10^6^ cells). Then the subsequent steps were performed as the procedure in cells.

### Statistical analysis

The data were presented as means ± SD. The results were analyzed by Student’s t test or one-way ANOVA using SPSS 22.0. *p* < 0.05 was considered as statistically significant.

## Results

### ATF6 attenuates the HFD induced hepatic steatosis by inducing H_2_S accumulation

**Fig. 1:**
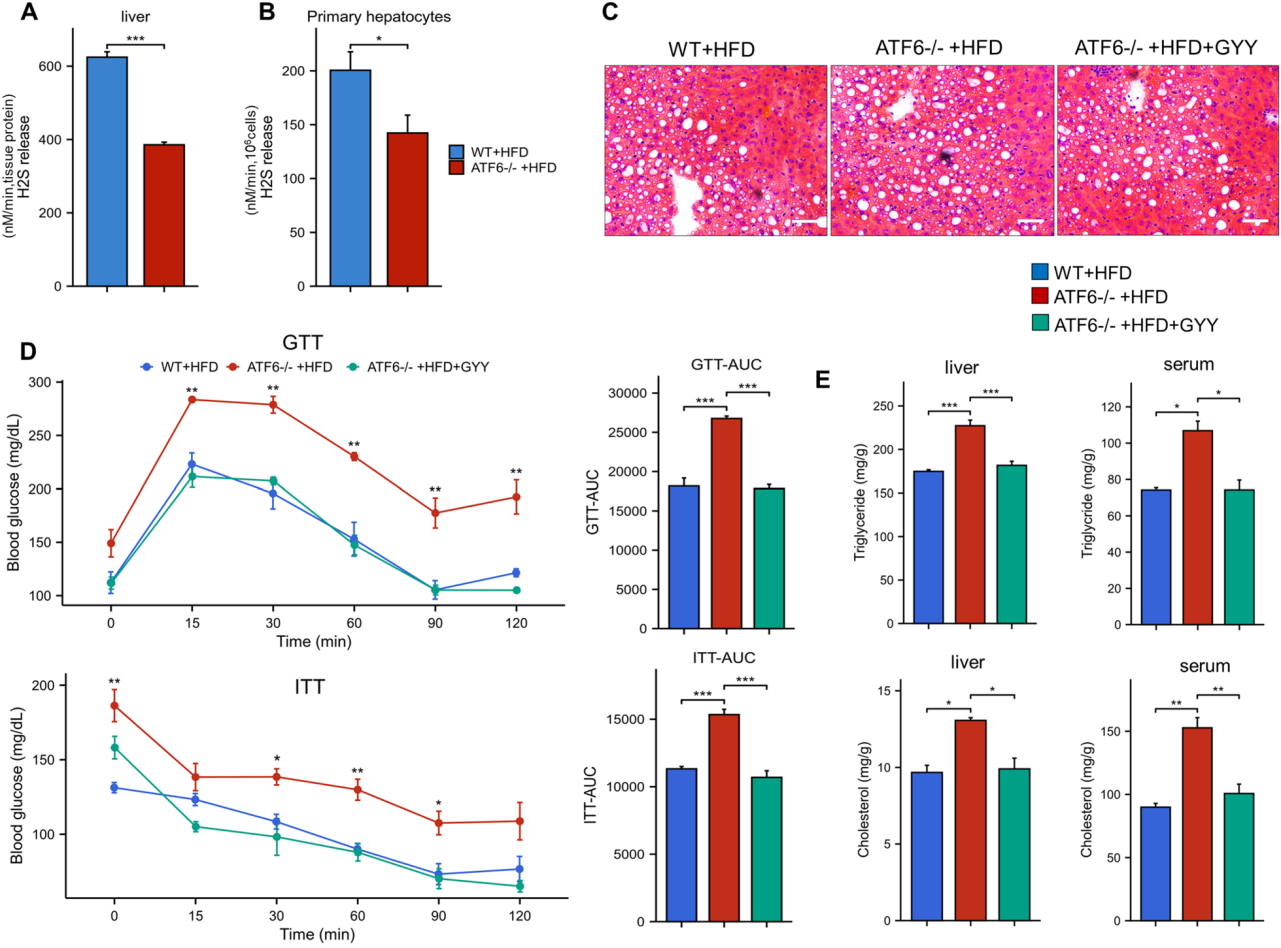
ATF6 attenuates hepatic steatosis in fatty liver by inducing H_2_S accumulation. **A** H_2_S level was analyzed in liver tissue of WT and ATF6^−/−^ mice fed on HFD. **B** H_2_S level was analyzed in primary mouse hepatocytes. **C** Histology analysis of liver in WT, ATF6^−/−^, and GYY4137 treated mice detected by HE stain (*200). **D** Glucose tolerance tests (GTT) and insulin tolerance tests (ITT) were performed with/without GYY4137 treatment in ATF6^−/−^ mice on HFD. **E** Liver and serum triglyceride and cholesterol levels in ATF6^−/−^ mice with/without GYY4137 treatment. The asterisks (*) marked in the graph represents the significant differences (p* < 0.05; p** < 0.01; p*** < 0.001) in different groups. (n = 3–4 for each group)

Previous studies showed that both ATF6 and H_2_S could moderate the fatty liver [7, 10]. In order to investigate the interaction between ATF6 and H_2_S in regulation of HFD induced fatty liver, the H_2_S level was examined in liver tissue in WT and ATF6^−/−^ mice fed on HFD. The results showed that the H_2_S level was significantly decreased in ATF6^−/−^ mice compared with WT mice (Fig. 1A), suggesting that the knockout of ATF6 reduces the H_2_S production. Subsequently, the same result was observed in primary mouse hepatocytes (Fig. 1B). Liver histology analysis suggested that ATF6^−/−^ mice showed liver steatosis fed with HFD (Fig. 1C). In our previous study, we already have demonstrated that the liver specific knockout of ATF6 affects the glycolipid metabolism [7]. In current study, ATF6^−/−^ mice showed glucose intolerance and impaired insulin sensitivity (Fig. 1D, E). To verify the effect of H_2_S on liver metabolism, we explore whether GYY4137, the slow-releasing H_2_S donor, could improve the state of glucose and lipid metabolism, and adipose accumulation on ATF6^−/−^ deficiency mice model in HFD condition. As shown in Fig. 1D, after the treatment with GYY4137, the blood glucose level is significantly decreased and the glucose tolerance is improved. Accordingly, the serum and intracellular hepatic concentrations of triglyceride (TG) and total cholesterol (TC) are dramatically decreased compared to control mice (Fig. 1E). Taken together, these results supported the hypothesis that liver specific knockout of ATF6 exacerbates liver metabolic damage by reducing the endogenous H_2_S production.

### ATF6 promotes CBS expression

**Fig. 2:**
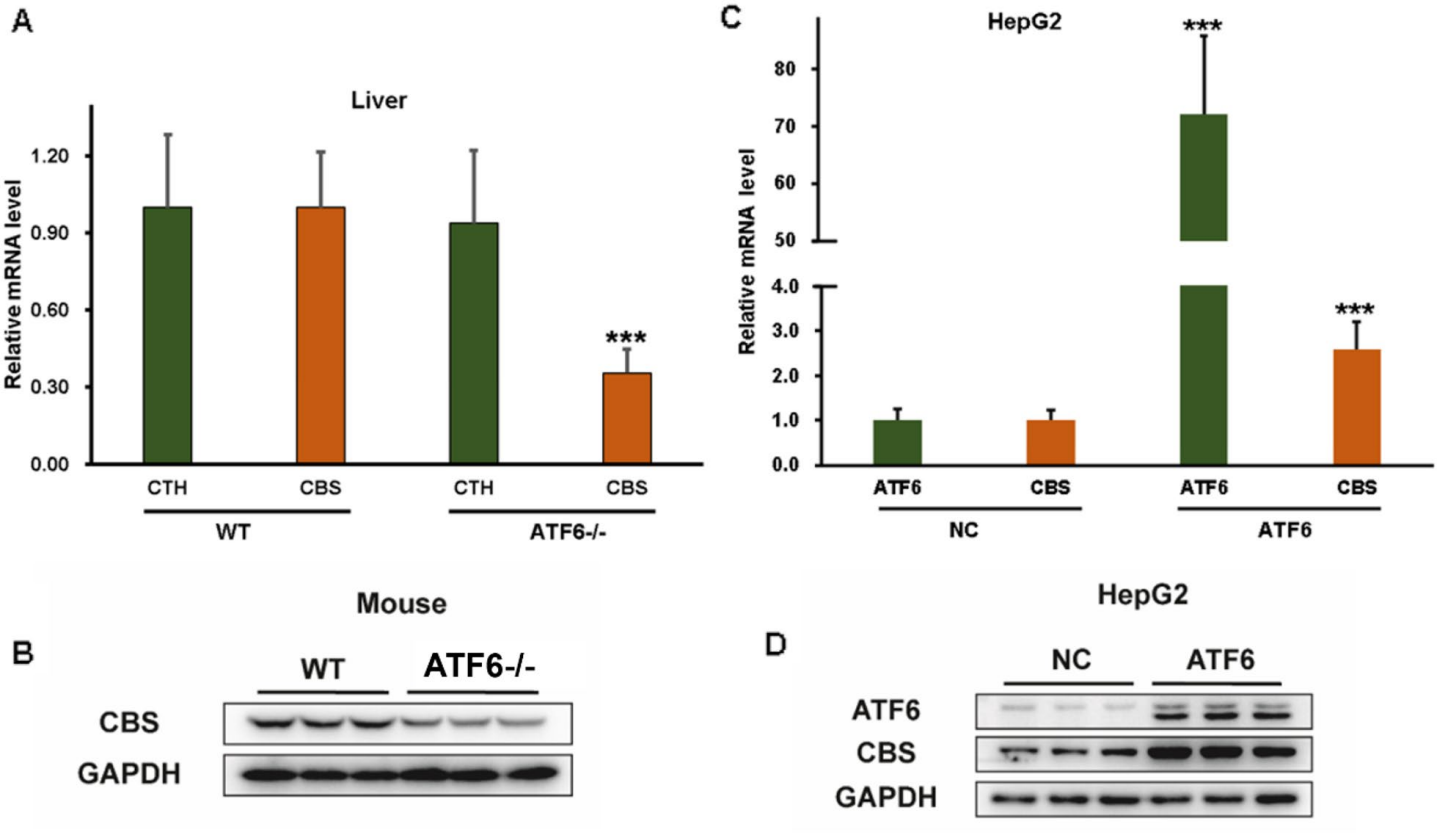
ATF6 upregulates CBS expression. **A** The mRNA levels of CBS and CTH were analyzed by qPCR in liver tissues from WT and ATF6^−/−^ mice fed with HFD. **B** The expression of CBS and CTH were evaluated by western bolt in liver tissues from WT and ATF6^−/−^ mice. **C** The mRNA levels of CBS were analyzed by qPCR in HepG2 cells with ATF6 overexpression and control ones, respectively. **D** The expression of CBS was evaluated by western blot in HepG2 cells with ATF6 overexpression and control ones. The asterisks (***) marked in the graph represents the significant differences (p*** < 0.001) in two groups. (n = 5–6). *WT* wildtype, *NC* negative control

CBS and CTH are the key H_2_S-metabolism enzymes in liver. First, we investigated the expression of CBS and CTH in ATF6^−/−^ mice and WT mice on HFD, to explore the association between ATF6 and H_2_S-gererating enzymes. There was an obviously decreased mRNA level of *CBS* in liver in ATF6^−/−^ mice compared with WT mice, while the CTH showed no significant differences between two groups (Fig. 2A). Subsequently, we also observed the similar result that CBS expression was markedly decreased in liver of ATF6^−/−^ mice by western bolt (Fig. 2B). To further verify the effect of ATF6 on CBS, we constructed the ATF6 overexpression vector and then transfected into HepG2 cells. As expected, the mRNA levels of CBS were increased about 2.5 times along with the ATF6 overexpression. The western blot result also confirmed the same result (Fig. 2C, D). Thus, ATF6 may upregulate the expression of CBS, but the exact molecular mechanisms need further investigation.

### ATF6 upregulates the promoter activity and expression of the *CBS* gene

**Fig. 3:**
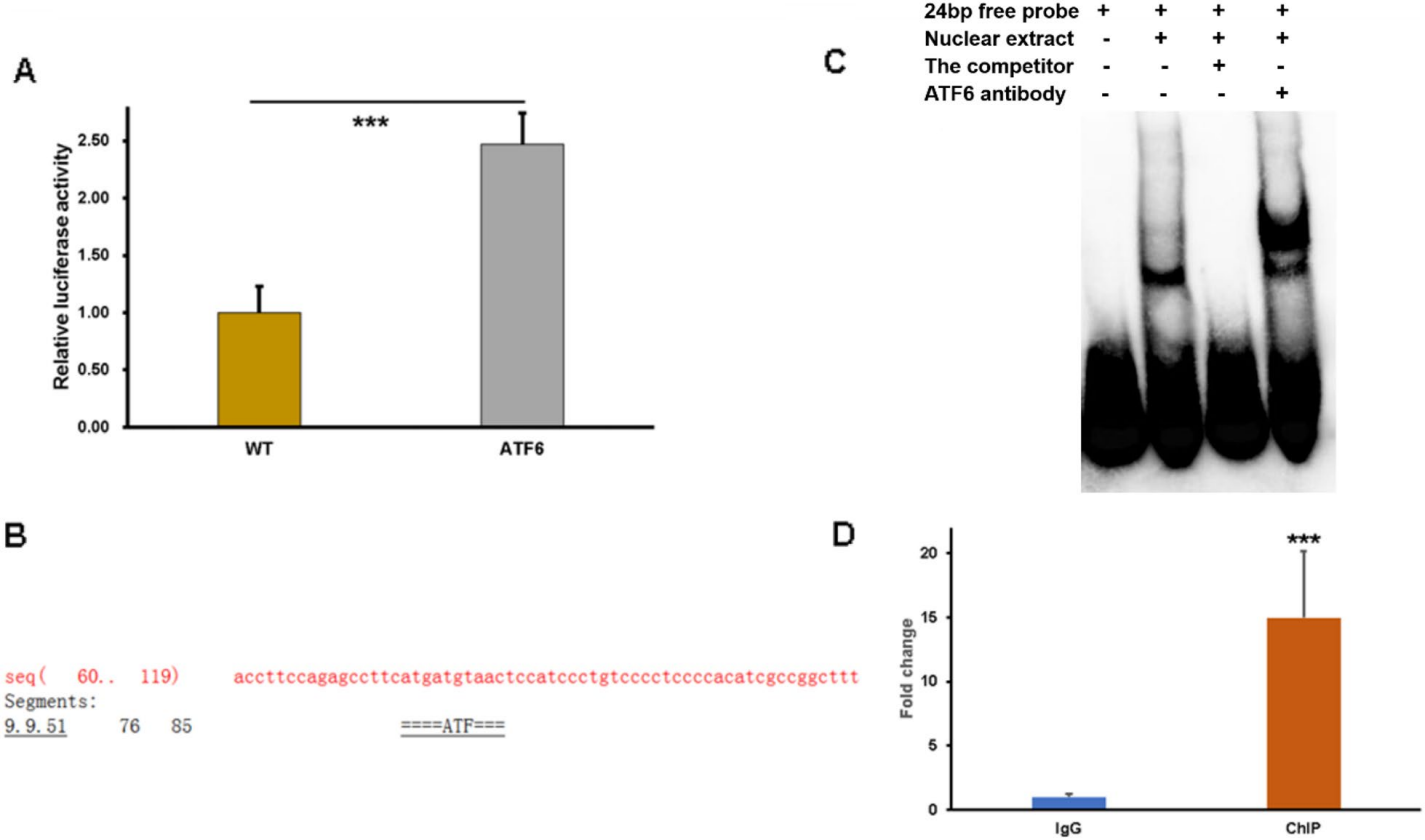
ATF6 upregulates the promoter activity and expression of the *CBS* gene. **A** HepG2 were co-transfected with the *CBS* promoter luciferase plasmids and ATF6 plasmids. Luciferase assays of *CBS* were performed. **B** The binding sites of ATF6 was predicted by AliBaba 2.1. **C** EMSA assays was carried out to explore the Binding affinity of ATF6 to biotin-labeled probe of *CBS*. **D** The ChIP-qPCR assay was performed to detect the ATF 6 enrichment level on the *CBS* promoter. The asterisks (***) marked in the graph represents the significant differences (p*** < 0.001) in two groups

As a transcription factor, ATF6 can promote the transcription of downstream target genes to exert its function. Considering that ATF6 upregulates the expression of CBS, we proposed that ATF6 might affect CBS expression at transcriptional level. To examine the molecular mechanism, the *CBS* promoter PGL3 plasmids were constructed. HepG2 cells were co-transfected with a luciferase reporter construct harboring a human *CBS* gene promoter and *ATF6*. The results showed that overexpression of ATF6 enhance the luciferase activity of *CBS* promoter (Fig. 3A), indicating that ATF6 may bind directly with *CBS* promoter to force its expression. Furthermore, the prediction by online software AliBaba 2.1 (http://gene-regulation.com/pub/programs/alibaba2/index.html) shows that ATF6 possesses the binding motifs in the promoter region of *CBS* (Fig. 3B). Subsequently, the EMSA experiment was conducted to further explore whether ATF6 bind to *CBS* promoter. ATF6 contained in nuclear extracts specifically bound to the biotin-labeled 24 bp probe to form the protein-DNA complexes, while the excess unlabeled oligonucleotide competitor competed with the binding site (Fig. 3C). In addition, for the binding reaction with the specific antibodies against ATF6, the super shift belt was observed. Finally, the ChIP-qPCR was performed to support the results (Fig. 3D). Taken together, we conclude that ATF6 could enriched on the promoter regions of *CBS*. These results indicate that *CBS* was the downstream target gene that upregulated by ATF6.

### ATF6 promotes Sirtuin-1 (SIRT1) sulfhydration

**Fig. 4:**
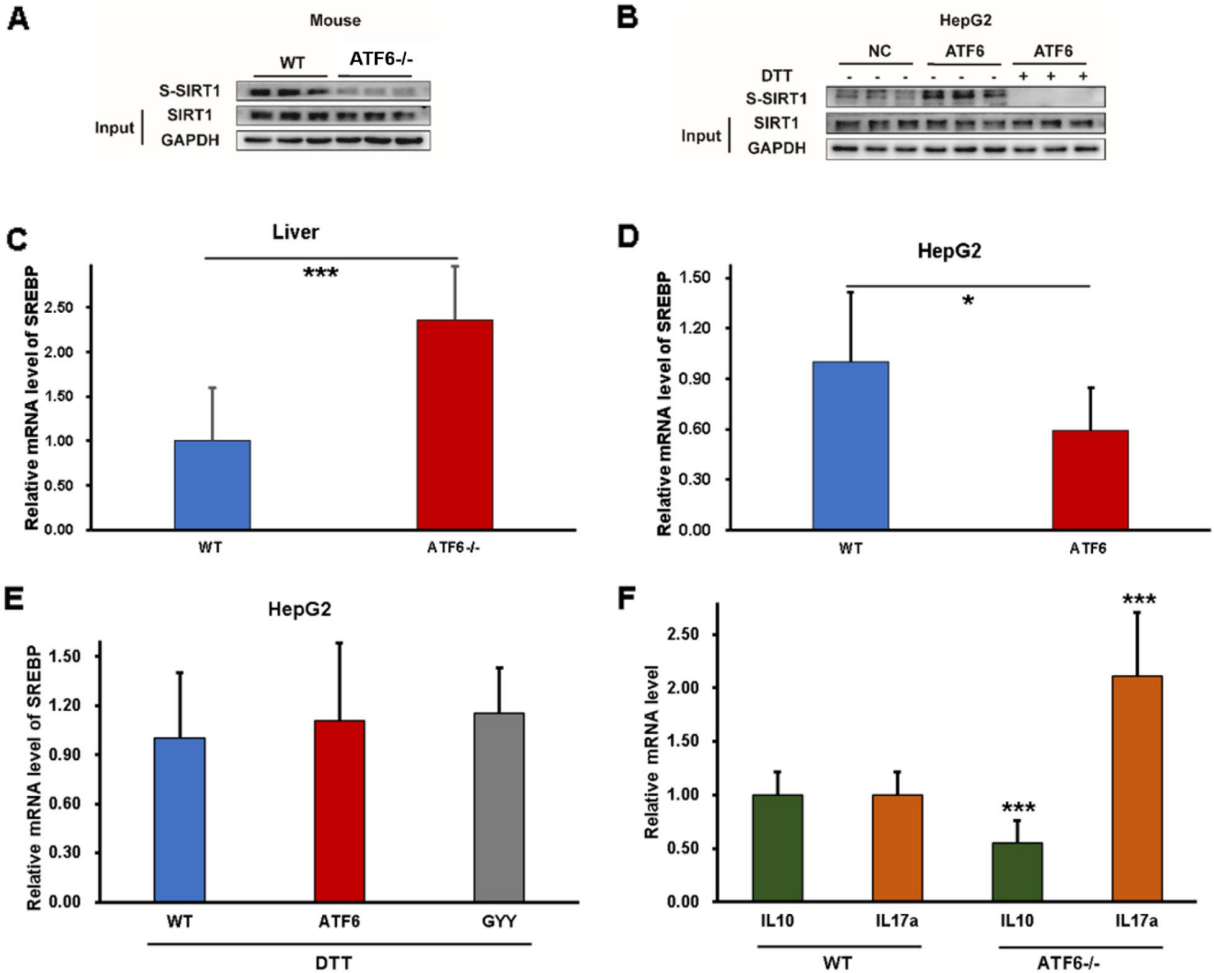
ATF6 can promotes SIRT1 sulfhydration by H_2_S signal and regulate the inflammation response by mediating IL-10/IL-17A. **A** Effect of ATF6 on the protein expression of SIRT1 in vivo. **B** Effect of ATF6 on the protein expression of SIRT1 in vitro. **C** Effect of ATF6 on the mRNA expression of SREBP in ATF6^−/−^ mice. **D** Effect of ATF6 on the mRNA expression of SREBP in HepG2 with ATF6 overexpression. **E** SREBP mRNA expression with DTT treatment. **F** The ATF6 effect on inflammatory cytokines. The asterisks (*) marked in the graph represents the significant differences (p* < 0.05; p*** < 0.001) in different groups. (n = 9 for each group). *WT* wildtype, *NC* negative control

It was reported that SIRT1, as an essential regulator in hepatic lipid metabolism, could be direct sulfhydrated by H_2_S [22, 23]. The sulfhydrated SIRT1 was assessed in vitro and in vivo in present study by modified biotin switch (S-sulfhydration) assay to investigate the effect of ATF6 on *SIRT1* signal. As shown in Fig. 4A, the sulfhydrated SIRT1 was significantly reduced in ATF^−/−^ mice. The upregulated sulfhydrated SIRT1 was observed in HepG2 cells with ATF6 overexpression. To explore the effect of H_2_S on SIRT1, the de-sulfhydration reagent, dithiothreitol (DDT) was used. The results showed that the sulfhydration was blocked after DDT pre-administration (Fig. 4B). Taken together, the results indicated that ATF6 may induce the *SIRT1* sulfhydration by H_2_S signal.

**Fig. 5:**
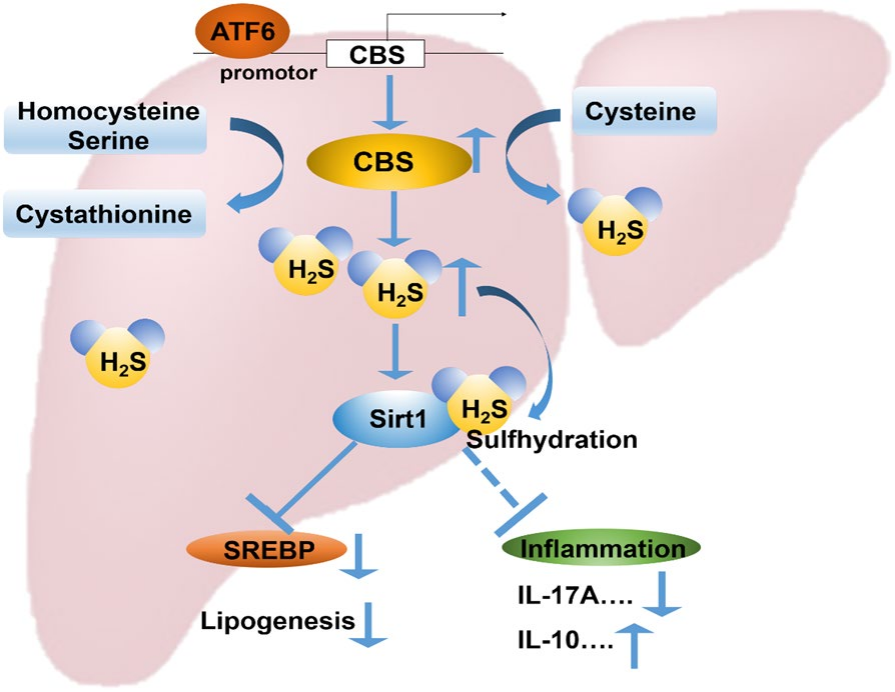
ATF6 increase Sirt1 sulfhydration by promoting H_2_S production through upregulating CBS expression. ATF6 binds to the promotor region of *CBS,* a vital H_2_S producing enzyme in the liver, and promote the protein transcription. The upregulation of CBS enhances H_2_S production. Accumulation of H_2_S stimulates Sirt1 sulfhydrylation, then inhibits its downstream SREBP expression, to reduce lipogenesis and fatty acid beta-oxidation. In addition, Sirt1 sulfhydrylation decrease expression of pro-inflammatory factor IL-17A, and increase anti-inflammatory factor IL-10 expression. ATF6 could mitigate the liver lipogenesis and inflammation by stimulating sulfhydrylated Sirt1 through CBS/H_2_S pathway

The sterol regulatory element-binding proteins (SREBP1c), as the downstream gene of SIRT1, has been proven to be the essential target of SIRT1 in regulating lipid metabolism [23]. Thus, we further investigated whether the SREBP1c expression was changed along with ATF6 levels. The results showed that the mRNA levels of *SREBP1c* increased in ATF^−/−^ mice compared with WT mice, while overexpressed ATF6 reduced *SREBP1c* mRNA levels (Fig. 4C, D). Subsequently, with DTT pretreatment to eliminate sulfhydration effect on SIRT1, there were no significant difference of *SREBP1c* mRNA levels between WT group, ATF6 overexpression group and GYY4137 co-incubation group (Fig. 4E). These data consistently showed that ATF6 could induce SIRT1 sulfhydration by H_2_S signal. Inflammatory cytokines play an important role in regulating liver lipid metabolism [24]. To explore the effect of ATF6 on cytokines and inflammation environment, the mRNA expression of the anti-inflammatory cytokine IL-10 (Interleukin-10) and pro-inflammatory cytokine IL-17A (Interleukin-17A) were tested in vivo subsequently. As shown in Fig. 4F, the anti-inflammatory cytokine IL-10 was downregulated, while the pro-inflammatory cytokine IL-17A was upregulated in ATF6 knockout mice, which indicated that ATF6 might regulate the inflammation response in liver (Fig. 5).

## Discussion

ATF6 act as the essential ER stress responsive regulator. Three key molecules involved in the ER stress-induced adaptive response, including IRE1α (endoribonuclease inositol-requiring enzyme 1-alpha), PERK (protein kinase RNA-like endoplasmic reticulum kinase) and ATF6 [2]. ER stress is recognized by those essential regulators, and then activate the downstream signal cascade. The major ER chaperone binding-immunoglobulin protein (BIP) binds to the ER-oriented parts of PERK and IRE1α. Active PERK phosphorylates the eukaryotic translation initiation factor 2 (eIF2), resulting in the selective inhibition of translation, effectively reducing ER client protein load. IRE1α autophosphorylates and activates its endonuclease domain, resulting in the cleavage of Xbp-1 to generate a shortened Xbp-1 isoform, which drives the production of various ER chaperones to restore ER homeostasis [25]. Upon activation by dissociation of BIP, ATF6 translocates into the Golgi apparatus where it is cleaved into the functional form. ATF6 can be regulated by XBP1, suggesting interactions between the IRE1α and ATF6 branches of the UPR, then increase ER capacity, and regulate cell survival [26].

ATF6 play vital role in a variety process of physiological and pathological conditions. ATF6 decreases myocardial ischemia/reperfusion damage and links ER stress and oxidative stress signaling in the cardia myocytes [26]. In the cellular model of inflammatory bowel diseases, ATF6 increases expression of inflammatory cytokines CXCL1 and tumor necrosis factor (TNF) in response to ER stress [27].

ATF6 may attenuate HFD-induced fatty liver and hepatic steatosis, and it has been verified that liver specific knockout of ATF6 exacerbated HFD-induced hepatic steatosis and glucose intolerance leading to liver metabolic damage [7]. Hepatic specific deficiency of ATF6 exacerbates liver metabolic damage by repressing autophage through mTOR pathway [7]. ATF6 attenuate hepatic steatosis by increasing fatty acid oxidation through peroxisome proliferator-activated receptor α (PPAR α) [28]. Thus, investigating the mechanism of ATF6 influencing liver metabolism may be helpful for the liver disease treatment. In the present study, we concluded that ATF6 could medicate the CBS/H_2_S signals by sulfhydrating SIRT1 to ameliorate the liver inflammation*.* However, the deep molecular mechanism by which ATF6 attenuates hepatic steatosis and glucose homeostasis need further investigation in the future.

H_2_S has been widely recognized as a novel gasotransmitter exerting physiological, regulatory or modulatory effects in mammalian cells and tissues by sulfhydration [9, 29]. H_2_S plays a key role in the process of autophagy in a variety of cells and tissues. In cardiomyocytes, H_2_S inhibits autophagy through the PI3K/SGK1/GSK3β signaling pathway [30]. In the model of renal ischemia–reperfusion injury, H_2_S inhibits autophagy via mediating scavenger receptor A signaling pathway, thereby improving endothelial cell dysfunction [31]. CBS, as the vital H_2_S-producing enzyme, participates in balancing the production and elimination of H_2_S. It has been reported that H_2_S may exert S-sulfhydrylation on various proteins by targeting the cysteine residues [32]. Accumulating evidences identified that SIRT1, which plays a vital beneficial role in liver lipid metabolism by regulating lipogenesis and fatty acid β-oxidation, could be sulfhydrated by H_2_S to promote its activity [22, 23]. Here, we question whether ATF6 interacts with CBS to exert its effect on H_2_S production, which further affect the SIRT1 activation by sulfhydrylation. In this study, we observed that the ATF6 indeed affects the SIRT1 sulfhydrylation. Specifically, after treatment with DDT, the sulfhydrylation of SIRT1 was completely blocked. Thus, ATF6 may induce the SIRT1 sulfhydrylation by CBS/H_2_S pathway. In addition, SIRT1, as a vital metabolic sensor, can downregulate the expression of SREBP1, a transcription factor participating in lipid synthesis [22]. In present study, we found that ATF6 exhibited negative regulation on SREBP mRNA expression. Especially, after treatment with DTT, the effect of ATF6 on SREBP disappeared. These results suggest that ATF6 may decrease SREBP expression by SIRT1 sulfhydrylation. Previous study showed that ATF6 attenuated SREBP2-related lipogenesis. Glucose deprivation caused the proteolytic cleavage of ER stress transducer ATF6, and the cleaved ATF6 translocated into nucleus binding to SREBP2, and recruited HDAC1, thus, the SREBP2-mediated lipogenesis and gene transcription were downregulated [33]. In addition, ER Stress Induces cleavage of ATF6 by the same proteases that process SREBPs ATF6 is processed by site-1 protease (S1P) and Site-2 protease (S2P), the enzymes processing SREBPs in response to cholesterol deprivation. [34]. In our study, further investigation was needed to clarify the molecular mechanism by which ATF6 regulates SREBP expression through SIRT1 sulfhydrylation.

Liver is not only an essential metabolic organ, but also an important immunological activity site. Accumulating evidence showed that there was a crosstalk interaction between inflammatory response and ER stress in liver disease [22]. Recent study showed that inhibition of ER stress ameliorated the inflammation in liver ischemia reperfusion injury [35]. In the acute liver injury, ATF6 upregulated macrophage-derived cytokines IL-1α expression and promoted liver fibrogenesis [36]. As the ER stress responsive gene, whether ATF6 act on the inflammatory mediation was explored. Our results suggested that ATF6 deficiency downregulated the anti-inflammatory cytokine IL-10, while upregulated the pro-inflammatory cytokine IL-17A expression in mice model. Thus, ATF6 may ameliorate inflammation by regulating the inflammatory cytokines in the fatty liver. The cytokines most commonly associated with hepatic disorders are the pro-inflammatory cytokines TNF-α, IL-1, IL-6, and transforming growth factor (TGF)-β. Those cytokines are also metabolic signals trigger this transit, where it is cleaved by S1P and S2P processing enzymes [37]. In the ischemia–reperfusion injury due to the metabolic stress in liver, the production of pro-inflammatory cytokines increased, while anti-inflammatory cytokine decreased. ATF6 altered the responsiveness against Toll-like receptor (TLR) stimulation during this process, and mediates a pro-inflammatory synergy [38].

There are several limitations and shortcomings of the present study. First, in this study, we demonstrated that GYY4137, the H_2_S releasing agent, ameliorated the glucose intolerance and lipid metabolism abnormalities on ATF6^−/−^ mice fed with HFD, suggesting that ATF6 attenuates hepatic steatosis in fatty liver by inducing H_2_S accumulation. However, we did not investigate the effect of GYY4137 on WT mice fed on normal diet or HFD. Second, our results indicated that ATF6 promote SIRT1 signal and regulate the inflammatory response in liver steatosis. Several inflammatory factors are involved in the process of inflammatory response in fatty liver. The investigation of anti-inflammatory factor IL-10 and pro-inflammatory factor IL-17A was limited. Third, we suggested that ATF6 downregulated the expression of SREBP via SIRT1. However, the molecular mechanism of regulation was unclear. Further investigation is needed to clarify those issue.

Continued on the previous study, we identified that ATF6 ameliorates liver steatosis and inflammation by upregulating CBS expression and H_2_S synthesis via SIRT1 sulfhydration. It would provide new insight for the development of potential therapeutic strategy for fatty liver.

## Conclusion

In conclusion, ATF6 increases SIRT1 sulfhydration by promoting H_2_S production through transcriptionally upregulating CBS expression, meanwhile it ameliorates the inflammation by regulating the inflammatory cytokines in the liver.

## Data Availability

The datasets analyzed in the current study are available from the corresponding author on reasonable request.

## Abbreviations

ATF6: Activating transcription factor 6
UPR: Unfolded protein response
GTT: Glucose tolerance test
ITT: Insulin tolerance test
CBS: Cystathionine β synthetase
H_2_S: Hydrogen sulfide
SIRT1: Sirtuin-1
ER: Endoplasmic reticulum
